# Dissociable neural systems of sequence learning

**DOI:** 10.2478/v10053-008-0105-1

**Published:** 2012-05-21

**Authors:** Freja Gheysen, Wim Fias

**Affiliations:** 1 Department of Experimental Psychology, Ghent University, Belgium

**Keywords:** sequence learning, neural systems, hippocampus, basal ganglia, serial reaction time task, artificial grammar task

## Abstract

Although current theories all point to distinct neural systems for sequence learning, no consensus has been reached on which factors crucially define this distinction. Dissociable judgment-linked versus motor-linked and implicit versus explicit neural systems have been proposed. This paper reviews these two distinctions, yet concludes that these traditional dichotomies prove insufficient to account for all data on sequence learning and its neural organization. Instead, a broader theoretical framework is necessary providing a more continuous means of dissociating sequence learning systems. We argue that a more recent theory, dissociating multidimensional versus unidimensional neural systems, might provide such framework, and we discuss this theory in relation to more general principles of associative learning and recent imaging findings.

## Introduction

A fundamental characteristic of human cognition is the ability to learn sequence information and to adapt to the environment based on this newly acquired knowledge, reflecting the remarkable plasticity of the human brain. Sequence knowledge is crucial for efficient daily functioning and therefore, omnipresent during life (Clegg, DiGirolamo, & Keele, 1998). For instance, in the morning we get dressed, go down the stairs, quickly reach for a cup, pour some coffee, drive the car and then start working on the computer. Imagine how inadequate our life would be when these actions, or parts of these actions, were performed in a different serial order. Frequently, the sequential regularities composing these actions are acquired through repeated practice and are often difficult if not impossible to describe, suggesting that sequence knowledge can be acquired in a procedural and unconscious way (Destrebecqz & Cleeremans, 2001; Stadler & Frensch, 1998). Sequence learning thus provides an intriguing example of implicit skill learning. Moreover, the apparent ease with which these skills can be performed is extremely remarkable because most of these actions actually entail complex sequence structures. Computer skills such as typing require a complex coordination of visuo-motor components which need to be sequenced properly in time.

It should therefore come as no surprise that the neural basis of sequence learning has interested researchers for many years. Numerous studies used imaging techniques such as positron emission tomography (PET; e.g., Destrebecqz et al., 2005; Honda et al., 1998; Peigneux et al., 2000; Rauch et al., 1995) and functional magnetic resonance imaging (fMRI; e.g., Forkstam, Hagoort, Fernandez, Ingvar, & Petersson, 2006; Lieberman, Chang, Chiao, Bookheimer, & Knowlton, 2004; Rauch et al., 1997; Schendan, Searl, Melrose, & Stern, 2003; Seger, Prabhakaran, Poldrack, & Gabrieli, 2000; Seidler et al., 2005; Skosnik et al., 2002; Thomas et al., 2004) to study the neural activation underlying sequence learning, or transcranial magnetic stimulation (TMS) to explore the causal involvement of several brain areas (e.g., Torriero, Oliveri, Koch, Caltagirone, & Petrosini, 2004; Udden et al., 2008). Neural regions including motor, prefrontal, parietal, temporal, occipital cortex, hippocampus, striatum, and cerebellum have been reported to be involved in sequence learning.

Current theories on learning all converge on the idea that the human brain does not learn in a unitary fashion but consists of multiple, dissociable neural systems operating on specific types of information (Fanselow, 2010; Squire, 2009). In the domain of sequence learning, no consensus has been reached on which factors crucially define the distinction of these neural networks. The present paper reviews and discusses two major distinctions the field has revolved around, that is, the distinction between judgment-linked vs. motor-linked and implicit vs. explicit neural networks. Instead of giving a complete and exhaustive literature overview, this paper highlights the discrepancies reported in the sequence learning literature which suggest that these strict traditional dissociations cannot fully account for all data. Ultimately, this review paper aims at reconciling these inconsistencies by describing another theory which crosses the judgment-linked versus motor-linked and implicit versus explicit learning frameworks: the theory of multidimensional versus unidimensional learning systems.

## Judgment-linked versus motor-linked neural systems

Sequence learning paradigms differ considerably with respect to the dependent measures that are used to assess sequence knowledge: Judgment-linked and motor-linked measures have been dissociated (Seger, 1997, 1998). *Judgment-linked tasks* measure sequence knowledge based on participants’ ability to make correct judgments about the stimulus sequences; a model task is the artificial grammar (AG) learning task (Reber, 1967). In a typical instantiation of this task, series of letter strings (e.g., *VXVS*) are presented that are constructed according to a finite-state grammar. This grammar represents a rule system defining the serial order in which letters can follow each other. In the learning phase, participants have to observe and memorize these meaningless letter strings. In a subsequent test phase, they are asked to judge whether new strings conform to the grammar or not. Successful, better-than-chance judgment suggests that participants have acquired sequence knowledge (Knowlton & Squire, 1996; Reber, 1967; Seger, 1998). On the other hand, *motor-linked* tasks assess sequence knowledge via the reaction time (RT) of motor responses, that is, the extent to which motor responses become more facilitated to sequenced stimuli compared to random stimuli. The most studied task that measures sequence learning via RT performance is the serial reaction time (SRT) task (Nissen & Bullemer, 1987). In a basic SRT task, a visual stimulus appears at one of the four horizontally aligned positions on a computer screen. Participants have to react as fast and as accurately as possible to the location of the stimulus by pressing the spatially corresponding key. The succession of the stimuli (and hence responses) follows a repeating sequential pattern. With continued practice, RTs become much faster on trials following the sequence than on trials violating the sequence. The RT differences between sequenced and non-sequenced (random) trials suggest that participants have learned the sequence. The SRT paradigm is an optimal task to study sequence learning given the relatively simple experimental implementation, the typically fast acquisition of sequence knowledge and the objective (RT) measurements to assess sequence learning (Clegg et al., 1998).

It has been suggested that tasks using judgment-linked versus motor-linked measures involve independent learning mechanisms relying on different areas in the brain, with the basal ganglia supporting the latter but not the former type of learning (Seger, 1997, 1998). On a behavioral level, Seger (1997, 1998) demonstrated functional dissociations between both forms of learning using the AG and SRT task. Several experimental manipulations (e.g., length of learning phase, assignment of sequence elements to screen location, letter set transfer) led to dissimilar or opposite effects on the judgment and motor linked measures of sequence knowledge. Neuropsychological research on Huntington‘s disease (HD) and Parkinson‘s disease (PD) patients has shown to be consistent with the view of dissociable judgment-linked and motor-linked sequence learning tasks. In these patient populations, preserved learning is found on the AG task (Knowlton et al., 1996; Peigneux, Meulemans, Van Der Linden, Salmon, & Petit, 1999; Reber & Squire, 1999; Smith, Siegert, & McDowall, 2001) while impaired learning is reported using the SRT task (Jackson, Jackson, Harrison, Henderson, & Kennard, 1995; Knopman & Nissen, 1991; Wilkinson, Khan, & Jahanshahi, 2009; Willingham & Koroshetz, 1993). These findings suggest that the basal ganglia might be involved only in motor- but not in judgment-linked sequence learning tasks. Along the same line, neuroimaging research stresses the role of the basal ganglia during SRT learning (Destrebecqz et al., 2005; Grafton, Hazeltine, & Ivry, 1995; Peigneux et al., 2000; Rauch et al., 1997), whereas AG learning studies report key functions for occipital and frontal areas of the brain (Seger et al., 2000; Skosnik et al., 2002; Udden et al., 2008).

However, results from other neuropsychological and imaging studies question such a strict dissociation. When modified versions of the judgment-linked AG and the motor-linked SRT task were used to more thoroughly investigate sequence learning abilities of patients with Parkinson’s disease, different results have been obtained. Impaired AG learning has been reported in a group of PD patients when using an AG task in which grammaticality judgments were made already during the learning phase and in which learning depended on trial-by-trial feedback learning (Smith & McDowall, 2006). On the other hand, preserved SRT learning was found using a verbal version of the SRT task (Smith et al., 2001); potentially because sequence learning abilities were less prone to the motor execution problems (e.g., bradykinesia, akinesia, tremor, rigidity) which characterize Parkinson’s disease. However, since another study using a verbal SRT task reported impaired sequence learning in subjects with PD (Westwater, McDowall, Siegert, Mossman, & Abernethy, 1998), Smith and colleagues (2001) argued that other factors might influence sequence learning results in PD patients such as the extent to which other brain structures (e.g., frontal lobe, cerebellum) are implicated in the disease and the severity and/or duration of the illness. Recent studies indeed demonstrated an association between sequence learning abilities of PD patients and the progress of the disease (known to be related to a greater fronto-striatal dysregulation): Increased general cognitive impairment (Vandenbossche, Deroost, Soetens, & Kerckhofs, 2009) and increased motor impairment (Wilkinson et al., 2009) were related to worse sequence learning abilities.

These recent data from neuropsychological studies thus seem to indicate that a strict dissociation between sequence learning mechanisms assessed through judgment-linked and motor-linked tasks is difficult to hold; sequence learning abilities of patients with striatal dysfunctions are also dependent on numerous aspects of group characteristics and methodology. Moreover, no clear double dissociations in neuropsychological research have been reported to date that supports a judgment-linked versus motor-linked dichotomy. Additionally, results from recent functional imaging studies challenge the idea that only motor-linked measures of sequence learning are reliant on the basal ganglia. Indeed, an important function for the caudate nucleus has been reported in imaging studies using AG (Forkstam et al., 2006; Lieberman et al., 2004) and other judgment-linked sequence learning tasks (Turk-Browne, Scholl, Chun, & Johnson, 2009).

Thus, while functional dissociations between tasks using judgment and motor-linked dependent measures have been demonstrated behaviorally (Seger, 1997, 1998), results at a neural level are not univocal. Moreover, it must be noted that these different sequence learning tasks do not only differ in the use of their dependent measures but also in other important aspects such as the phase of the learning process and the modality of sequence information.

### Early versus late sequence learning phase

The process of sequence learning typically involves several successive stages, going from an early, fast learning phase with strong performance improvements to a later phase of automatization and consolidation, which rely on different areas in the brain (Doyon et al., 2009). As such, a clear interpretation of differential activation between AG and SRT imaging studies remains difficult since it is likely that they tap different phases of the learning process: With AG learning tasks brain activation is generally measured during the sequence judgments in the test phase (e.g., Forkstam et al., 2006; Lieberman et al., 2004; Seger et al., 2000; Skosnik et al., 2002) while SRT studies typically collect fMRI data online during the learning phase (e.g., Peigneux et al., 2000; Schendan et al., 2003; Seidler et al., 2005; Thomas et al., 2004). Brain activation in the former studies therefore involves recollection of the sequence knowledge, while the latter studies typically collect brain activation beginning from the earlier acquisition stage.

### Perceptual versus motor sequence information

AG learning is generally considered to reflect higher-order perceptual learning while SRT learning is viewed as motor skill learning (Seger, 1998; Smith et al., 2001). Within the implicit learning literature, extensive behavioral research has been concerned with separating perceptual sequence learning (learning of stimulus-stimulus associations, i.e., not involving response related associations) from motor related sequence learning (learning of response-response, response-stimulus or stimulus-response associations) and has explored their differences using modified versions of the SRT task (e.g., Deroost & Soetens, 2006; Gheysen, Gevers, De Schutter, Van Waelvelde, & Fias, 2009; Howard, Howard, & Mutter, 1992; Mayr, 1996; Nattkemper & Prinz, 1997; Remillard, 2003; Willingham, 1999). These studies have suggested that implicit perceptual sequence learning is more vulnerable than motor-related sequence learning; they showed that learning perceptual sequence information can be enhanced by using simple sequence structures (Deroost & Soetens, 2006) or salient stimulus material (Kelly, Burton, Riedel, & Lynch, 2003) and reflects a slower acquisition process than comparable implicit motor related learning (Gheysen et al., 2009). In neuroimaging research, however, potential perceptual-motor differences have largely been ignored. A previous fMRI study has attempted to address this issue using a transfer SRT task (Bischoff-Grethe, Goedert, Willingham, & Grafton, 2004). Participants first performed an SRT task with a spatially incompatible stimulus-response mapping, subsequently, they transferred to a compatible condition in which either the response sequence (motor transfer group) or the stimulus sequence (perceptual transfer group) was retained. However, since participants in the perceptual transfer condition showed no evidence of learning the sequence of stimulus locations, the neural activation between modalities could not be meaningfully compared.

In recent fMRI studies, we specifically focused on the question whether separate neural networks are specialized for perceptual (non-motor) related versus motor related implicit sequence learning or, alternatively, whether these different modalities share a common network (Gheysen, Van Opstal, Roggeman, Van Waelvelde, & Fias, 2010, 2011). We used a novel paradigm, the serial color matching task, which allows studying both perceptual and motor sequence learning using identical visuo-motor and cognitive demands. In this task, participants were instructed to match the colors of three small squares with the color of a subsequently presented large target square. In the perceptual version of the task, the sequence structure was assigned to the succession of target colors while in the motor version of the task, the sequence structure was linked to the succession of finger responses. A meaningful comparison between both learning processes was possible because identical task demands, materials, sequence structure, procedure, and statistical analyses were used in both studies. Moreover, to take different stages of the sequence learning process into account, we tracked both the behavioral and neural time course of learning across two scanning sessions with additional sequence training (outside the scanner) in between. Behaviorally, the motor sequence learning process developed considerably faster than the perceptual sequence learning process but both processes occurred in a comparably implicit way. The comparison of imaging results between perceptual and motor learning was focused on two brain areas: the caudate nucleus and the hippocampus. The caudate nucleus displayed learning dependent activation during the second scanning session in a similar and gradual pattern for both perceptual and motor sequence information. This finding is in accord with imaging studies showing an important contribution of the striatum in both perceptual based AG learning (Forkstam et al., 2006; Lieberman et al., 2004) and motor based SRT learning (Destrebecqz et al., 2005; Peigneux et al., 2000; Rauch et al., 1997). Interestingly, a crucial activation was found in a comparable area of the right anterior caudate nucleus in our task using motor-related dependent measures (Gheysen et al., 2011) and in AG learning tasks using judgment-based dependent measures (Forkstam et al., 2006; Lieberman et al., 2004). For both types of tasks, the head of the caudate nucleus correlated positively with sequence knowledge (Forkstam et al., 2006; Gheysen et al., 2011). The hippocampus, on the other hand, displayed sequence learning related activation earlier in the learning process (first scanning session). Furthermore, the sequence learning-related activation in the hippocampus was more pronounced under motor related compared to perceptual (non-motor) related learning conditions.

Likewise, in the context of intentional motor skill learning, there is also ample evidence indicating that the learning phase and modality of sequence representation must be taken into account to fully understand the neural organization of sequence learning (for reviews, see Doyon et al., 2009; Hikosaka et al., 1999; Hikosaka, Nakamura, Sakai, & Nakahara, 2002). According to the neurobiological model of Hikosaka and colleagues (2002), a new motor skill is first encoded in spatial coordinates and is then gradually represented into a motor coordinate system. These early and advanced phases of motor skill learning depend on two dissociable cortico-striatal circuits. Early in learning, the anterior/associative part of the striatum interacts with fronto-parietal cortices in order to acquire an accurate, spatial representation of the sequence. With practice, there is a shift of activation towards the posterior/sensorimotor part of the striatum. This region cooperates with motor cortical areas in order to build a long-term motor representation of the sequence (Coynel et al., 2010; Lehéricy et al., 2005).

## Implicit versus explicit neural systems

For a long time, consciousness has been regarded as a crucial factor dissociating the neural networks of learning and memory (Shanks & St. John, 1994; Squire, 1992, 2009; Tulving, 1987). The idea that implicit (unconscious) sequence learning is neurally independent of explicit (conscious) sequence learning partly comes from neuropsychological studies on amnesic patients (suffering from a dysfunction of the medial temporal lobe/hippocampus) and PD patients (suffering from a basal ganglia dysfunction due to damage of dopaminergic cells in the substantia nigra). A traditional theory in the field of learning and memory is that the hippocampus and basal ganglia systems are divided by consciousness, with the hippocampus subserving explicit learning and the basal ganglia subserving implicit forms of learning (Squire, 2009). Using the SRT task, some studies indeed demonstrated that amnesic patients with hippocampal lesions retain the ability to learn sequences despite having no explicit knowledge (e.g., Gagnon, Foster, Turcotte, & Jongenelis, 2004; Nissen & Bullemer, 1987; Reber & Squire, 1994) whereas implicit SRT learning has been found to be impaired in PD patients (Jackson et al., 1995; Siegert, Taylor, Weatherall, & Abernethy, 2006; Wilkinson et al., 2009).

Yet, these findings are not consistent. Using more complex sequence material in the SRT task, Curran (1997) demonstrated that amnesic patients do not learn higher-order sequence information as efficiently as controls do. Likewise, Vandenberghe and colleagues (2006) showed impaired implicit sequence learning in amnesic patients with a more complex, probabilistic sequence. These latter results are consistent with other studies showing impaired implicit learning in patients with amnesia (e.g., Chun & Phelps, 1999; Ryan, Althoff, Whitlow, & Cohen, 2000). Conversely, implicit sequence learning in PD patients has been shown to be only moderately impaired (Ferraro, Balota, & Connor, 1993; Shin & Ivry, 2003) or even intact (Smith et al., 2001), and findings of impaired explicit sequence learning in PD patients have been reported as well (Ghilardi, Eidelberg, Silvestri, & Ghez, 2003; Wilkinson et al., 2009). Taken together, the results from prior sequence learning studies on amnesic and PD patients suggest that the traditional explicit-hippocampus and implicit-basal ganglia framework is probably too simplistic and that other factors need to be taken into account to fully understand the distinct function of both neural structures to sequence learning.

Besides neuropsychological studies, also neuroimaging research has extensively focused on the question whether implicit and explicit sequence learning networks can be dissociated. Several studies specifically investigated whether brain activation differs depending on the extent to which participants gained awareness of the sequence information using different approaches. For example, Grafton and colleagues (1995) contrasted SRT learning with and without an attentional distraction task (tone counting) to capture brain activation under respectively implicit and explicit learning conditions. Brain activation differed under both conditions: Implicit learning involved motor cortex, supplementary motor cortex, and putamen whereas explicit learning recruited right dorsolateral prefrontal cortex, right premotor cortex, and biparieto-occipital cortex. Similar findings of different neural networks were reported in a subsequent study using comparable single and dual task conditions (Hazeltine, Grafton, & Ivry, 1997), although in the explicit (single task) condition, a more ventral set of areas were engaged (comprising inferior occipital, temporal, and frontal cortex) probably related to the use of color instead of spatial stimuli.

Other studies used single (SRT) task paradigms contrasting the neural responses before and after participants became aware of the presence of a sequence structure (Honda et al., 1998; Rauch et al., 1995). As such, Honda and colleagues (1998) found comparable results as the studies from Grafton et al. (1995) and Hazeltine et al. (1997) in that frontoparietal areas were predominantly responsible for explicit learning and more central areas (e.g., primary somatosensory areas) for implicit learning. Rauch and colleagues (1995) reported somewhat different brain areas comprising the distinct neural networks: Implicit learning involved the right ventral premotor cortex, the right ventral caudate/nucleus accumbens, the right thalamus, and the extrastriate cortex whereas explicit learning involved the primary visual cortex, the perisylvian cortex, and the cerebellum.

Destrebecqz et al. (2005) used another approach to study the neural differences between implicit and explicit forms of sequence learning. They scanned participants during a sequence recall task with the process dissociation procedure (PDP). The PDP has been introduced by Jacoby (1991) for the study of implicit memory but has been adapted by Destrebecqz and Cleeremans (2001) to sequence learning. This method assesses the relative contribution of implicit and explicit processes during sequence learning by asking participants to first generate a sequence that resembles the trained sequence as much as possible, and subsequently, to generate a sequence that differs as much as possible from the trained sequence. The degree with which consciousness contributes to sequence learning can be measured by computing the difference between generation performance under inclusion and exclusion instructions. By correlating these measures with the regional cerebral blood flow, Destrebecqz et al. (2005) found that the anterior cingulate cortex/medial prefrontal cortex and the striatum were differentially involved in sequence learning, in that the first region supported explicit knowledge and the latter supported implicit knowledge.

Other imaging studies, however, challenge the idea that independent brain networks are involved in implicit and explicit sequence learning and report no clear dissociation. For instance, Schendan and colleagues (2003) contrasted an implicit learning condition (in which participants were naive about the presence of a repeating sequence) with a subsequent explicit learning condition (in which participant received prior sequence knowledge). They demonstrated that implicit and explicit learning activated largely overlapping neural areas including the medial temporal lobe, the striatum, and the dorsolateral prefrontal cortex. Overlapping activation was also noted by another study which randomly alternated implicit and explicit learning conditions (Willingham, Salidis, & Gabrieli, 2002). They reported activation in a common neural network of left prefrontal cortex, right putamen, and left inferior parietal cortex. In accordance with these latter two studies, Aizenstein and colleagues (2004) reported comparable activation in prefrontal cortex, striatum, anterior cingulated cortex, and visual cortex when presenting an implicit and explicit sequence simultaneously, by using colored shapes and assigning color and shape to different, independent sequences.

In sum, we can conclude that previous results on the issue whether dissociable brain systems are involved in implicit and explicit sequence learning are far from univocal: Some studies report non-overlapping brain activations while others report considerably overlapping neural networks. The interpretation of these contradictory results is complicated by the fact that different sequences were used (e.g., deterministic and probabilistic sequences) with different amounts of prior sequence knowledge in the explicit learning conditions. It is also questionable whether the differential activation between conditions is not related to differential attentional requirements (e.g., when contrasting single vs. dual task performance) or to time-related factors (e.g., when contrasting performance before and after participants become aware of the sequence). Moreover, as argued by Destrebecqz and colleagues (2005), another factor might hamper a correct interpretation of previous results. Starting from experimental conditions which are a priori considered to involve exclusively implicit and exclusively explicit learning processes is risky since this exclusiveness assumption does not necessarily hold. For instance, it has been demonstrated that, although SRT studies are designed to induce implicit sequence learning, participants develop sequence awareness (as in e.g., Howard et al., 1992; Willingham, Nissen, & Bullemer, 1989).

## Multidimensional versus unidimensional neural systems

Although these previous frameworks have provided useful insights in how neural networks of sequence learning might be dissociated, the literature overview indicates that the judgment versus motor linked and the implicit versus explicit dichotomy prove insufficient. It appears that a broader theoretical framework is necessary to explain the various findings on sequence learning and its neural organization. One such framework has been proposed by Keele, Ivry, Mayr, Hazeltine, and Heuer (2003). Their theory posits that the human brain supports two broad systems of sequence learning: a multidimensional and a unidimensional system.

The term *dimension* refers to a specific type or modality of sequence information. Our daily environment involves a continuous stream of various types of sequential information coming from multiple sources. For instance, when riding home from work, we coordinate our limbs in a sequential pattern to move forward and, simultaneously, we process the succession of visual and auditory information surrounding us. As argued by Keele et al. (2003), the term *dimension* can be used to describe distinctions within a system (e.g., hands vs. feet in the motor system or color vs. shape in the visual system). As such, the multidimensional system is proposed to build associations between events from these multiple dimensions (e.g., motor, visual, and auditory information) whereas the unidimensional system is restricted to the association of information along a single dimension (e.g., only visual information). Evidence for this theory stems largely from studies contrasting performance during single and dual SRT task conditions. In the latter condition, successive stimuli typically alternate between two dimensions, that is, the spatial information from the primary SRT task and the auditory information from the secondary tone-counting task (e.g., Curran & Keele, 1993; Grafton et al., 1995; Schmidtke & Heuer, 1997). Based on these data, Keele and colleagues (2003) concluded that the two systems typically differ in their attentional constraints and their susceptibility to context information. The unidimensional sequence learning system forms associations between events, being attended or not. Moreover, since this system only associates information within one dimension, this type of learning cannot be disrupted by context information. The multidimensional system, on the other hand, can be influenced by context information since it naturally integrates all incoming information across dimensions. Crucially, this system is restricted in that only attended (task relevant) information gains access. If this information includes correlated events, associations among these events will be formed, yet, if uncorrelated events are introduced, cross-dimensional learning will be disrupted. Indeed, Schmidtke and Heuer (1997) reported different effects on sequence learning when participants transferred from a single to a dual SRT task condition using interleaved random or sequenced auditory tones. When visual stimuli alternated with random tones (as is typically the case in dual task settings), sequence learning became less pronounced in reference to the single SRT task. However, when using correlated sequenced tones in the dual SRT task, sequence learning was comparable to what was found in the single SRT task. It was therefore argued that the unidimensional system is always involved in both single and dual task conditions and is responsible for the preserved sequence learning in a dual task setting with random interleaving tones. The multidimensional system, on the other hand, is more limited in that its function during dual task conditions depends on whether or not the attended context information is correlated.

On a neural level, Keele and colleagues (2003) claimed that the multidimensional system involves ventral pathways for sequence learning including temporal and lateral prefrontal cortex, whereas the unidimensional system engages more dorsal circuits including parietal and motor cortex. This dissociation in terms of neural organization stems from several imaging studies contrasting single and dual SRT task performance (e.g., Grafton et al., 1995; Hazeltine et al., 1997), with the single SRT task condition being assumed to reflect the additional contribution of the multidimensional system. Regarding subcortical brain involvement, the theory proposed different roles for the basal ganglia and the hippocampus, two neural systems well known for their function in sequence learning (Albouy et al., 2008; Schendan et al., 2003): Whereas the hippocampal system was hypothesized to operate exclusively in multidimensional learning, the basal ganglia were linked to both unidimensional and multidimensional sequence learning.

### Relation to general principles of associative learning

Interestingly, the critical distinction made by Keele and colleagues (2003) between a sequence learning network with and without the capability of cross-dimensional association, fits well with general principles of associative learning and memory (Colgin, Moser, & Moser, 2008; O’Reilly & Norman, 2002; O’Reilly & Rudy, 2001). An important line of research posits that the hippocampal learning system is important for *conjunctive learning* (or variously described as *configural* or *contextual learning*); for combining detailed information from multiple cortical streams into a unified representation rather than for processing simple, elemental information (for a review, see O’Reilly & Rudy, 2001). This idea stems, in part, from the ubiquitous finding in conditioning research on rodents that hippocampal lesions critically disrupt conditioning to multimodal cues (e.g., cues containing a specific combination of temporal, visual, and spatial information) but not to unimodal cues (Iordanova, Burnett, Aggleton, Good, & Honey, 2009; Phillips & LeDoux, 1992). Likewise, recent fMRI studies in humans have suggested that, if information has to be integrated across different modalities, hippocampal activation is to be expected (Staresina & Davachi, 2009; Tendolkar et al., 2007). Neuroanatomical data are compatible with this theory; they indicate that the hippocampus is particularly suited for cross-dimensional binding since it receives information from virtually all cortical association areas (Dickerson & Eichenbaum, 2010; Suzuki & Amaral, 2004).

This conjunctive learning framework accords well with the theory of *pattern separation*. The hippocampus has been suggested to serve as a pattern separator which makes the representations of similar events more distinguishable by decorrelating these events and remapping them unto non-overlapping representations (for a review, see Colgin et al., 2008). This notion has mainly emerged from insights of so-called place cells in the rodent brain. Place cells are neurons in the hippo-campus that are activated whenever an animal moves in a specific location in space. It was demonstrated that the firing rate in place cells alter following only minor changes of the environment, reflecting the formation of new, distinct memory representations that do not overlap with prior representations of similar environments (Colgin et al., 2008). In the context of sequence learning, such mechanism might be very advantageous since it reduces interference between similar conditions (e.g., the four response conditions in a classical SRT task) and hence can facilitate the association process between these conditions. Furthermore, the function of the hippocampus as a pattern separator is compatible with the principle of cross-dimensional integration. The richer the information projected unto the hippocampus, the more it needs to be integrated and remapped unto a unitary, sparse representation (Atallah, Frank, & O’Reilly, 2004; O’Reilly & Rudy, 2001). While the hippocampus has thus been claimed to be specialized for the rapid learning of detailed context information due to its highly conjunctive, pattern-separated representations; the basal ganglia have been suggested to work together with the frontal cortex in a slower learning process of more generalized information due to their highly overlapping representations (Attalah et al., 2004; O’Reilly & Norman, 2002).

Altogether, theories on associative learning have proposed rather dissociable functions for the hippocampus and the basal ganglia system. Our recent neuroimaging findings are consistent with this notion and fit quite well with the multidimensional/hippocampal learning framework. As mentioned above, we recently conducted two fMRI studies to investigate whether or not perceptual and motor sequence learning depend on different neural networks using a novel paradigm: the serial color matching task (Gheysen et al., 2010, 2011). Interestingly, we demonstrated that the caudate nucleus displayed a similar, gradual learning function for both perceptual and motor sequences, whereas the hippocampus reflected a much faster learning system which was more pronounced for the motor compared to the perceptual task. Overall, the hippocampus is not regarded as a specific motor system and has been repeatedly associated with perceptual forms of associative learning (e.g., Fortin, Agster, & Eichenbaum, 2002; Lieberman et al., 2004; Turk-Browne et al., 2009; Van Opstal, Verguts, Orban, & Fias, 2008). Moreover, given the fact that sequence learning during the perceptual and motor task occurred on a comparably implicit basis, the larger hippocampal contribution during the motor task cannot be ascribed to modality as such (being perceptual or motor related) or different levels of awareness. Instead, we argued that the multidimensional/hippocampal framework might give a reasonable explanation for our findings. Motor responses composing the sequence structure in the motor task entail information from multiple dimensions going from the obvious motor related information to proprioceptive, tactile, and spatial information whereas the colors composing the sequence structure in the perceptual task constitute elemental visual information. Potentially, the motor sequence learning condition triggered the hippocampal function more than the perceptual sequence learning condition since it naturally contained information from multiple dimensions. In contrast, the caudate activation in our studies did not differ for motor (multimodal) and perceptual (unimodal) sequence information, which is in accord with Keele et al.’s (2003) theory describing a role for the basal ganglia in both multi- and unidimensional sequence learning systems. Furthermore, the faster learning rate we found for the motor (hippocampal based) learning process is consistent with the model of O’Reilly and Rudy (2001) indicating a specific function for the hippocampus in the rapid encoding of conjunctive information.

### Relation to dissociable judgment versus motor-linked and implicit versus explicit learning systems

Differences between judgment and motor-linked sequence learning tasks might be understood within the multidimensional-unidimensional framework. In general, motor-linked tasks (such as the SRT task) are more sensitive to the multidimensional system than judgment-linked tasks. Sequence learning assessed through the SRT task naturally involves a mix of response, stimulus, and stimulus-response contingencies. On the other hand, judgment-linked sequence learning with the AG task only involves stimulus contingencies in the form of letter strings. The differential involvement of cross-dimensional sequence information, and thus the differential sensitivity for an engagement of the multidimensional system, might explain previous imaging findings. That is, an important function for the hippocampus has been reported in SRT sequence learning (Albouy et al., 2008; Schendan et al., 2003) while AG learning studies reported key functions for stimulus-specific visual cortical areas (Seger et al., 2000; Skosnik et al., 2002). The basal ganglia, on the other hand, have been reported to play a role in sequence learning for both judgment- (Forkstam et al., 2006; Lieberman et al., 2004) and motor-linked paradigms (Destrebecqz et al., 2005; Peigneux et al., 2000; Rauch et al., 1997), which is in accord with the theory of Keele et al. (2003) proposing a function for the basal ganglia in both uni- and multidimensional learning systems. Nevertheless, the relationship between both frameworks requires further direct investigations.

Interestingly, the multidimensional-unidimensional framework also offers a new perspective on the distinction between implicit and explicit learning (Keele et al., 2003). Since the unidimensional system is hypothesized to operate without attention, learning is more likely to occur on an implicit basis. Conversely, although learning within the multidimensional system can also occur implicitly, it is more prone to explicit sequence awareness because the information is attended. Indeed, Curran and Keele (1993) provided evidence for the two systems being differentially accessible by awareness. Single SRT task performance was compared to subsequent dual SRT task performance for both explicit and implicit learners. In the single task condition, sequence learning correlated positively with awareness: Although both groups showed evidence of sequence learning, aware participants learned more than unaware participants did. In the dual task, sequence learning persisted but was overall smaller than in the preceding single task. Interestingly, no correlation between awareness and sequence learning was found any more. This led to the conclusion that during single task performance, two sequence learning systems must operate in parallel: one (multidimensional) system which is accessible to awareness and another (unidimensional) system which is not dependent on awareness but remains operational during dual task learning.

Moreover, the inconsistent results from neuropsychological studies on amnesic patients with hippocampal damage might be understood within the multidimensional-unidimensional framework. Traditionally, it has been claimed that the hippocampus subserves explicit learning but not implicit learning (Squire, 2009) and indeed, several SRT studies reported intact sequence learning in amnesic patients despite having no explicit knowledge (e.g., Gagnon et al., 2004; Nissen & Bullemer, 1987; Reber & Squire, 1994). However, studies using more complex sequence structures demonstrated impaired implicit sequence learning in subjects with amnesia (Curran, 1997; Vandenberghe et al., 2006). Likewise, for an implicit contextual cuing task, impaired learning in amnesic patients has been reported (Chun & Phelps, 1999). Altogether, these data suggest that the implicit-explicit distinction is not sufficient to understand the hippocampal learning function; instead, the hippocampal system is important for conditions in which the integration of larger and more complex context information is necessary, even if this implies implicit learning situations.

## Conclusions

Research on the neural basis of sequence learning has led to the consensus that sequence learning is not unitary in nature but is mediated by distinct neural systems. However, how exactly these learning systems are divided remains unclear. A distinction has been proposed between sequence learning assessed through judgment-linked (e.g., artificial grammar) and motor-linked (e.g., SRT) tasks (Seger, 1997, 1998). However, neuropsychological and neuroimaging findings have not been straightforward, suggesting that other factors must be taken into account to fully understand the operating principles characterizing the distinct sequence learning systems. Another classical distinction, which has been proposed for more than two decades, is between implicit and explicit learning processes (Shanks & St.John, 1994; Tulving, 1987). Neuroscientists have linked this distinction frequently to basal ganglia versus hippocampal learning systems (Squire, 2009). In the field of sequence learning, the same distinction has been made; yet, results from neuroscience research have not been univocal and accumulating data show that this implicit-explicit distinction proves insufficient.

Altogether, we feel a more coherent framework is necessary that crosses previous dichotomies; a framework that uses a more continuous approach to characterize distinct learning systems. We have argued that another theory, dissociating multidimensional versus unidimensional sequence learning systems (Keele et al., 2003), might provide such framework and involves a richer means of explaining the wide range of behavioral and neural data on sequence learning. Ultimately, this model is of great interest since it highly relates with general principles of associative learning and memory (O’Reilly & Rudy, 2001). It remains a great challenge though, to further develop a coherent and computationally explicit theory on the neural organization of sequence learning.
